# Correlates of physical activity habits in adolescents: A systematic review

**DOI:** 10.3389/fphys.2023.1131195

**Published:** 2023-04-21

**Authors:** Tianyi Shao, Xiaogang Zhou

**Affiliations:** ^1^ College of Education, Zhejiang Normal University, Jinhua, China; ^2^ School of Educational Studies, Universiti Sains Malaysia, Penang, Malaysia

**Keywords:** adolescents, physical activity habits, physical factors, cognitive factors, lifestyle factors

## Abstract

Physical activity habits are crucial for the physical and mental wellbeing of adolescents (individuals aged 10–19 years). However, few studies over the last two decades have systematically aggregated the influential factors of physical activity habits for adolescents. Five online databases (EBSCOhost (Eric), Psychology and Behavioral Sciences Collection, PubMed, Scopus, Web of Science) were searched for relevant studies published before 14 August 2022. Our systematic review indicated the following: 1) boys performed more physical activity habits than girls, whereas girls preferred to engage in moderate-to-vigorous physical activity; 2) physical activity in adolescents decreased with age; 3) African American adolescents performed significantly more habitual physical activities than white adolescents; 4) adolescents with higher literacy had better physical activity habits; 5) support from parents, teachers, friends, and others helped adolescents in developing physical activity habits; 6) adolescents who spent less time participating in habitual physical activity had a higher body mass index; 7) adolescents who reported higher levels of self-efficacy and satisfaction with school sports tended to have stronger physical activity habits; 8) sedentary behavior, smoking, drinking, prolonged screen time, negative emotions, and excessive use of media technology were correlated with reduced habitual physical activity in adolescents. These findings could help develop interventions to motivate adolescents and promote physical activity habits among them.

## 1 Introduction

Over recent years, the promotion of physical activity behaviors in adolescents has become a recognized goal of public health agencies worldwide (PAGACS, 2018; PGAC, 2018 Physical Activity Guidelines Advisory Committee). Adolescence is a critical period of physical and mental growth, and lifestyle and health-promoting behaviors during this period are crucial (Patton et al., 2016). Therefore, the World Health Organization recommends adolescents to engage in regular physical activity, particularly moderate-to-vigorous physical activity (chiefly aerobic activities) for 60 min on average and high-intensity aerobic activity and muscle and bone strengthening activities on at least 3 days per week (Bull et al., 2020). Despite the numerous benefits of physical activity for adolescents, recent surveys have shown that the compliance rates for physical activity among adolescents are not promising. Data from a survey of 1.6 million participants from 146 countries (regions) indicated that more than 81% of adolescents worldwide failed to meet the recommended amount of physical activity (Guthold et al., 2020). Alarmingly, adherence to physical activity among adolescents tended to decline with age (Dumith et al., 2011), and several adolescents gradually discontinued physical activities. A previous study reported that 29.7% (n = 894) of 3013 10-year-old exercise participants dropped out of physical activity at the age of 12 years and that 33.3% (n = 705) of 2016 12-year-old exercise participants dropped out of physical activity at the age of 14 years (Vella et al., 2020).

Habits are often defined as repeatedly and frequently performed behaviors (Ouellette and Wood, 1998; Rhodes and Courneya, 2003). Habit formation relies on context-dependent repetition; that is, reliable and frequent initiation of a behavior in the same context is a prerequisite for the behavior to become a habit (Gardner et al., 2011). When applied to exercise psychology, physical activity habits are healthy lifestyle behaviors that can be performed consistently and frequently over a long period of time, which can be automatically elicited without considerable intentional effort (Aarts et al., 1997).

Research on physical activity habits did not become prevalent until the 21st century. In 2008, Verplanken and Melkevik applied the Self-Reported Habit Index for exercise behavior, and their initial study showed that this measure was stable and reliable, more importantly, confirmed that habits are different from motor behavior frequency, intention, and perceived behavioral control (Verplanken and Melkevik, 2008). The number of studies on physical activity habits in adolescents has increased substantially in recent years, and the findings show that various factors influence exercise habits (Hagger, 2019; Lee and Yoon, 2019). To date, only one review has examined the correlates of physical activity habits in adolescents; nonetheless, it was not strictly a systematic review and was published much earlier (Aarts et al., 1997).

Considering the need for an updated and comprehensive literature review on this topic, we therefore systematically reviewed the literature to identify the factors influencing the physical activity habits of adolescents.

## 2 Materials and methods

### 2.1 Literature sources and search strategy

This study adopted a two-step search strategy to identify relevant studies. Based on the PRISMA statement (Moher et al., 2015), studies were identified for inclusion through five electronic databases (EBSCO host (Eric), Psychology and Behavioral Sciences Collection, PubMed, Scopus, Web of Science). Our search strategy involved the following terms: 1) adolescent* OR teens OR teenager* OR juvenile OR school-aged AND children; 2) physical AND activity OR physical AND exercise OR sports AND activities OR sport AND movement OR sport*. activities OR sport OR movement OR sport* OR motor OR athletic AND sports; and 3) habit* OR custom* OR behavior AND habit* (see Supplementary Material S1 [Supplementary Table S1] for the search strategy used in each database).

All originally retrieved records were imported into the EndNote X9 data management system and were independently confirmed and managed by two authors. If any discrepancy arose in this process, the final decision was made through a consensus discussion.

### 2.2 Inclusion and exclusion criteria

The inclusion criteria were as follows: 1) studies on correlates of physical activity habits in adolescents; 2) studies with participants aged between 10 and 19 years, defined as “adolescents” by the World Health Organization (WHO, 2022), or those with a mean age within this range; 3) articles published in English; 4) peer-reviewed journal articles; and 5) empirical studies. Studies were excluded if they 1) were not related to the topic; 2) focused on unhealthy or special populations (e.g., individuals with chronic diseases or disabilities and professional athletes); 3) were non-empirical studies; 4) were published in a language other than English; or 5) were dissertations, conference articles and abstracts, reviews and correspondence, and unpublished articles.

### 2.3 Study quality evaluation and risk of bias

To assess the methodological quality of the included studies, we used an adaptation of the McMaster Review Scale-Quantitative Studies (Moher et al., 2015; Sarmento et al., 2018). This scale was used to assess the methodological quality of previous studies conducted in similar fields (Imms, 2008; Lu et al., 2017). The form contains 16 items, including study purpose (one item), study context (one item), study design (one item), sampling (two items), measurement (four items), data analysis (four items), conclusions (one item), and implications and limitations (two items). The methodological quality of each study was assessed by two independent reviewers, Prof. Jing Qi and Prof. Qidi Li, who thoroughly analyzed the relevant information. Through a comprehensive analysis, they evaluated the overall quality of the articles [Supplementary Table S2]. Each item was assigned with 1 point when it was clearly described and present) or 0 point when it was inadequately described or not present. Any uncertainties and disagreements were resolved by the authors. The total score for each study was calculated by adding the total scores of the relevant items and dividing them by the total possible score. Scores of <50%, 51%–75%, and >75% indicated low, good, and high quality, respectively (Zeng et al., 2017).

## 3 Results

**FIGURE 1:**
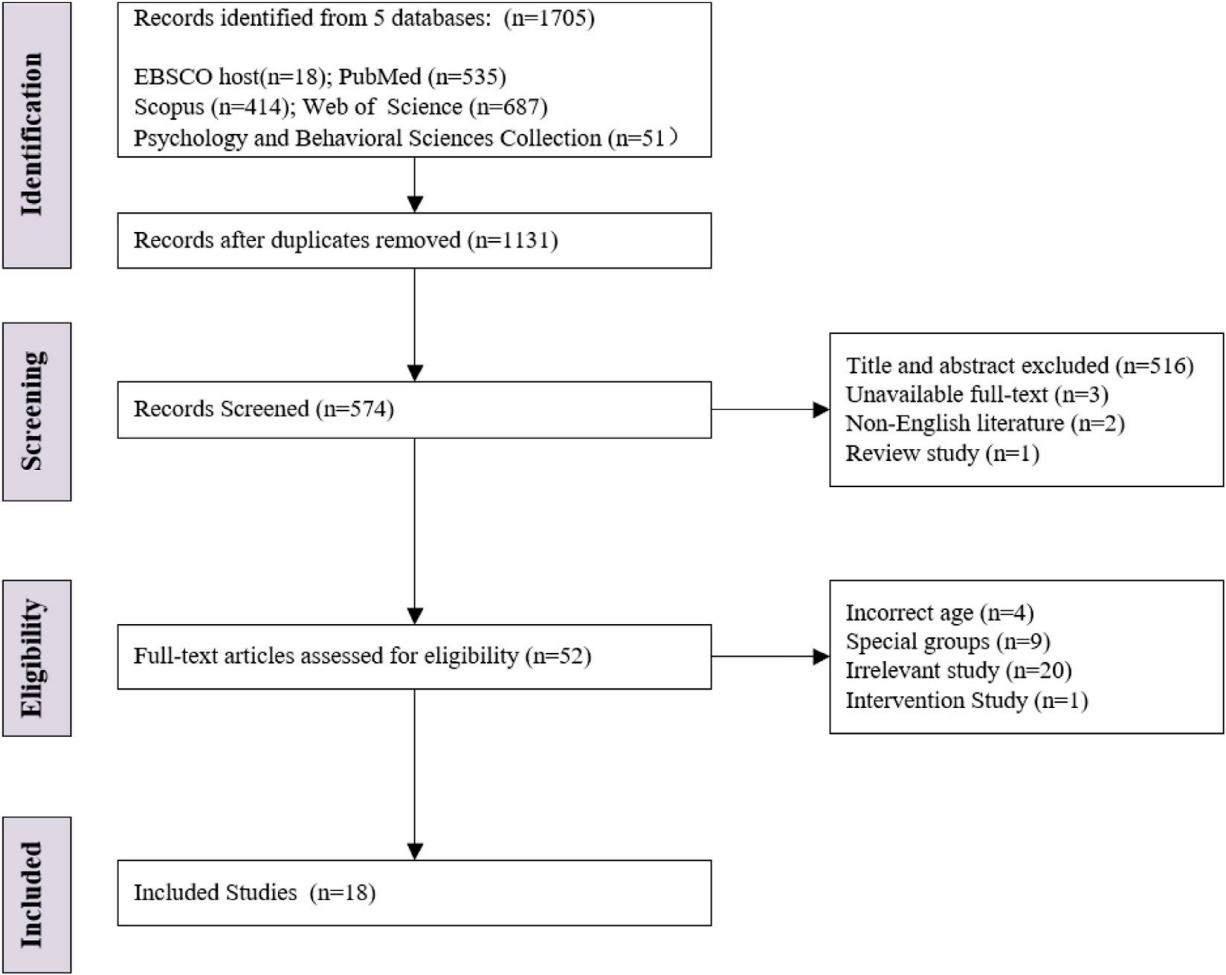
Flow diagram of the study selection process.

The initial database search yielded a total of 1705 publications. Removal of duplicates resulted in 1131 remaining articles. After screening the publications by title and abstract, 574 articles were obtained. After reading the titles and abstracts and ranking the articles with non-English full text and reviews, 52 articles were obtained initially. Subsequently, the full texts of the remaining articles were read, and 18 articles were finally included after excluding studies with age discrepancies, special populations, and interventions and irrelevant articles (Figure 1).

Comprehensive data extraction was performed for each study, including authors’ names and publication year, country of origin, focus, sampling characteristics (such as participant demographics, including age and sex, as well as sample size), study design, measurement tools, and main results. Data were extracted from each article using Microsoft Excel 2019. The key information of each study is presented in Table 1.

**TABLE 1:** Characteristics of the included articles.

Author/Year/Country	Focus	Sampling characteristic	Design	Measurement	Main results
Bénéfice et al. (2001) Senegal	To examine habitual physical activity and its association with growth and nutritional status in adolescents from rural Senegal	40 adolescents (Mean age = 13.3 ± 0.5)	Prospective	CSA accelerometers	The stature was negatively correlated with physical activity level, whereas the body mass index was positively associated pubertal status and subcutaneous fatness were not significant predictors of physical activity levels
Garnier and Benefice (2001) Senegal	To analyze the relationship between nutritional and maturational status and habitual physical activity	40 migrant and 40 non-migrant adolescents (Mean age = 13.4 ± 15.3)	Longitudinal	3-day survey	Less mature migrants are more physically active, and girls at the end of puberty reach a higher intensity index
Schmitz et al. (2002) USA	To explore the association of demographic and psychosocial factors with physical activity and sedentary leisure habits in adolescents	3798 adolescents aged 11–15 (Mean age = 12.8)	Cross-sectional	Two questions on a 5-point Likert scale	Physical activity and sedentary leisure habits were associated with race, academic rank values of health, appearance, and parenting style
Gavarry et al. (2003) France	To analyze habitual physical activity in adolescents from elementary to high school	182 adolescents aged 6–20	Cross-sectional	A questionnaire and a daily activity diary	Males experienced a significant decrease of 69% and females a decrease of 36% in total physical activity levels from elementary to high school
Klentrou et al. (2003) Canada	To examine the relationship between habitual physical activity, attitudes toward participation in physical activity, aerobic fitness, body fat and the frequency of upper respiratory tract infections in adolescents	256 adolescents (Mean age = 14.3 ± 0.3)	Cohort	The Habitual Activity Estimation Scale	Adolescents who participate in less physical activity showed a higher body fat percentage, lower aerobic fitness, and a higher frequency of upper respiratory tract infections
Kim et al. (2007) Korea	To examine the influence of psychosocial influences (social support, self-concept, and self-efficacy) on adolescents’ physical activity habits	3653 adolescents aged 16–19	Cross-sectional	Leisure-Time Exercise Questionnaire	Adolescents’ self-concept, self-efficacy, and support from parents, teachers, close friends, peers, and others contribute to improved physical activity levels
Kaya et al. (2010) Turkey	To investigate the physical activity habits of adolescents in a semi-urban area of Istanbul	369 adolescents aged 11–14 (Mean age = 12.5 ± 0.87)	Cross-sectional	Questionnaire	There are some sex-based differences in physical activity habits, with boys participating in physical activity more often than girls
Moreno-Murcia et al. (2011) Spain	To explore the influence of physical self-concept on physical activity and other healthy lifestyle habits	472 adolescents aged 16–20 (Mean age = 17.37 ± 0.95)	Cross-sectional	Habitual Physical Activity Questionnaire	Current physical activity is positively correlated with the willingness to engage in physical activity in the future and to adopt healthy lifestyle habits
Bąk-Sosnowska and Skrzypulec-Plinta (2012) Polish	To analyze the physical activity preferences of adolescents and compare adolescents’ lifestyle statements with their parents’ beliefs	711 adolescents aged 14–15	Cross-sectional	10-question survey	Physical activity levels were significantly correlated with the frequency of snack purchases
Hosseini et al. (2013) Iran	To explore the role of parents in the development of physical activity habits in adolescents	16 adolescents aged 10–19	Qualitative	Not mentioned	The role of parents in the physical activity of adolescent girls was to develop an interest in physical activity
Piéron and Ruiz-Juan (2013) Spain	To assess the relationship between the social environment and the physical activity habits of adolescents	6170 adolescents aged 12–16	Cross-sectional	Ad hoce questionnaire	Family environment has been identified as a risk factor of physical inactivity in adolescent, with students are less likely to exercise regularly if their family members have never been physically active
Zach et al. (2013) Israel	To present the result of physical activity habits among adolescents based on the national health survey	6274 adolescents from 7 to 12 grades	Cross-sectional	MABAT youth questionnaire	Middle school students were more physically active than high school students, boys were more physically active than girls, and Arab students were more physically active than Jewish students
Dos Santos et al. (2014) Mozambique	To explore the changing trends of habitual physical activity of Mozambican adolescents	3393 adolescents aged 8–15	Longitudinal	Questionnaire	The level of habitual physical activity among Mozambican adolescents exhibited a negative secular trend with age
Wafa et al. (2014) Malaysia	To examine objectively measured physical activity in Malaysian adolescents and to compare the differences in physical activity levels between obese and healthy weight individuals	86 obese adolescents and 86 healthy weight adolescents	Cross-sectional	Actigraph accelerometers	Physical activity levels were particularly low in healthy and obese individuals, but the obese group had significantly lower moderate-to-vigorous physical activity than healthy group
Wushe et al. (2014) South Africa	To explore the habitual physical activity of adolescents in the North West Province of South Africa by race and sex using an objective approach	226 adolescents aged 15–19	Observational cohort	Actiheart^®^	Habitual physical activity levels among South African adolescents vary by sex and race. Girls are more active than boys in moderate-to-vigorous physical activity, and white adolescents are more active than black adolescents
Kelishadi et al. (2016) Iran	To compare the dietary and physical activity habits of a nationally representative sample of Iranian adolescents according to their family and regional socioeconomic status	13,486 adolescents aged 6–18 (Mean age = 12.5)	Cross-sectional	World Health Organization Global School-based Student Health Survey	Total screen time, time spent working on the computer and watching television, and sedentary time was significantly higher for those with higher household socioeconomic status
López Sánchez et al. (2016) Spain	To analyze the habitual physical activity levels adolescents in the Murcia region	1055 adolescents (Mean age = 11.77 ± 2.86)	Cross-sectional	Physician-based Assessment and Counseling for Exercise questionnaire	There were sex-based differences in physical activity, with more active boys than active girls
Carayanni et al. (2021) Greece	To examine the effects of socioeconomic status, nutrition, physical activity habits, and perceptions on body mass index in Greek adolescents	5144 adolescents aged 12–15	Cross-sectional	Questionnaires	Significant associations were identified between nutrition, and physical activity habits, in addition to significant sex-based differences in sociodemographic and nutritional factors and physical activity habits

### 3.1 Demographic factors

Twelve studies examined the relationship between physical activity habits and demographic factors in adolescents (Schmitz et al., 2002; Gavarry et al., 2003; Kim et al., 2007; Kaya et al., 2010; Hosseini et al., 2013; Piéron and Ruiz-Juan, 2013; Zach et al., 2013; Dos Santos et al., 2014; Wushe et al., 2014; Kelishadi et al., 2016; López Sánchez et al., 2016; Carayanni et al., 2021).

#### 3.1.1 Sex

The studies consistently indicated sex-based differences in physical activity habits. Boys more frequently participated in physical activity, including outdoor sports activities, more frequently than girls, with a significant difference between both sexes (Kaya et al., 2010; Zach et al., 2013; Carayanni et al., 2021). A significant statistical difference was found in physical activity participation between male and female adolescents, with girls reporting significantly lower levels of participation than boys (Carayanni et al., 2021). Furthermore, adolescent girls spent more time in engaging in moderate-to-vigorous physical activity than adolescent boys (Wushe et al., 2014). A sex-related difference in physical activity levels was also observed between boys and girls, with more active boys (31.2%) than active girls (14.9%); additionally, boys performed physical activities on one more day per week than girls.

#### 3.1.2 Age

Gavarry et al. reported that the total physical activity decreased from elementary school to high school by 69% for boys and 36% for girls (Gavarry et al., 2003). Zach et al. indicated a clear negative correlation between physical activity levels and age in both boys and girls; those who were classified as inactive had a significantly higher mean age, whereas those who were sufficiently active exhibited the lowest mean age (Zach et al., 2013). Additionally, Dos Santos et al.(2014) revealed that physical activity habits among adolescents in Mozambique showed a decreasing trend with age.

#### 3.1.3 Race

Schmitz et al. (2002) identified a notable correlation between race and physical activity patterns, revealing that Caucasian students exhibited higher physical activity levels and lower levels of sedentary leisure habits than their peers of other races. White adolescents habitually engage in more moderate-to-vigorous physical activity than black adolescents (Wushe et al., 2014). Zach et al. (2013) found that Arab students exhibited a higher level of physical activity than did their Jewish counterparts.

#### 3.1.4 Cultural and socioeconomic factors

Schmitz et al. (2002) found that physical activity levels and sedentary leisure habits of adolescents may be influenced by cultural differences. According to Kelishadi et al.(2016), a higher socioeconomic status was associated with a greater screen time, particularly while working on computers and watching television, as well as more sedentary time.

#### 3.1.5 Family environment

Kim et al. (2007) suggested that physical activity levels can be influenced by support from multiple sources, including parents, teachers, friends, and classmates. Especially, the role of parents was crucial in the development of physical activity among adolescent girls, who helped them in developing an interest in initiating and sustaining physical activity (Hosseini et al., 2013). Conversely, another study contended that adolescents whose family members, specifically their parents and siblings, had never been physically active, were more likely to refrain from engaging in regular physical activity (Piéron and Ruiz-Juan, 2013).

The included studies were of high quality and based on the best evidence synthesis, strong evidence suggested that the above demographic elements were factors influencing the physical activity habits of adolescents.

### 3.2 Health factors

Six studies examined the relationship between adolescent physical activity habits and health factors, including body shape, and body fat (Bénéfice et al., 2001; Garnier and Bénéfice, 2001; Schmitz et al., 2002; Klentrou et al., 2003; Bąk-Sosnowska and Skrzypulec-Plinta, 2012; Wafa et al., 2014). Physical activity levels were negatively correlated with stature but were positively associated with the body mass index. Neither pubertal status nor subcutaneous fatness were found to be significant predictors of physical activity levels. Garnier and Bénéfice demonstrated that less mature immigrants were more physically active and that girls at the end of puberty (mature state) achieved a higher intensity index (Garnier and Bénéfice, 2001). Schmitz et al. (2002) found that girls who placed a higher value on their health, appearance, and achievement were more likely to engage in higher levels of physical activity. Klentrou et al. (2003) reported that those who spent less time in physical activity had a higher body fat percentage, lower aerobic fitness, and higher frequency of upper respiratory infections. Bąk-Sosnowska and Skrzypulec-Plinta. (2012) concluded that the least physically active students purchased the least snacks, whereas the most active students purchased the most snacks. According to Wafa et al. (2014) physical activity levels were low in both healthy and obese adolescents; however, individuals with obesity displayed significantly lower moderate-to-vigorous physical activity levels than healthy individuals. The described studies were all high-quality studies; based on the best-evidence synthesis, strong evidence suggests that the aforementioned health factors influence physical activity habits in adolescents.

### 3.3 Cognitive factors

Three studies examined the relationship between adolescent physical activity habits and cognitive factors (including perceptions of sport, self-concept, and body perception) (Schmitz et al., 2002; Kim et al., 2007; Moreno-Murcia et al., 2011). Schmitz et al. (2002) found that girls raised with authoritative parenting styles had higher physical activity levels and less sedentary leisure habits. Another study showed that adolescents with a positive self-concept and high self-efficacy for physical activity were more likely to engage in physical activity (Schmitz et al., 2002). Moreno-Murcia et al. (2011) revealed that physical self-concept (perceived motor ability, physical attractiveness) was positively related to an individual’s current physical activity. These studies were all high-quality studies; based on the best-evidence synthesis, strong evidence suggests that the above cognitive factors influence physical activity habits in adolescents.

### 3.4 Lifestyle factors

Three studies examined the relationship between adolescent physical activity habits and lifestyle factors (sedentary, smoking, and alcohol use, among others) (Moreno-Murcia et al., 2011; Bąk-Sosnowska and Skrzypulec-Plinta, 2012; Dos Santos et al., 2014). Lifestyle factors could play a crucial role in determining the physical activity levels in adolescents (Dos Santos et al., 2014). Moreno-Murcia et al. (2011) found that physical activity was negatively associated with tobacco and alcohol consumption. Another study identified a correlation between diary habits and adolescents’ physical activity engagement, and observed significant disparities in the frequency of school shop snack purchases among adolescents with varying levels of physical activity (Bąk-Sosnowska and Skrzypulec-Plinta, 2012). These studies were all high-quality studies; based on the best-evidence synthesis, strong evidence suggests that the aforementioned lifestyle factors influence physical activity habits in adolescents.

## 4 Discussion

This review systematically aggregated the correlates of physical activity habits in adolescents, highlighting the influence of demographic, health, cognitive, and lifestyle factors on physical activity habits in this population. The findings of this review were consistent with those of previous studies on factors influencing physical activity habits in adolescents.

First, differences in physical activity habits among adolescents of different sexes have been observed before (Armstrong et al., 2011; (Drenowatz et al., 2010; Kopcakova et al., 2014), which may be attributed to differences in the selection of physical activity, methods, and attitudes among adolescents of different sexes. Second, age plausibly plays a more notable role in shaping physical activity behaviors and that the likelihood of sustained participation in a particular activity decreases throughout adolescence (Bélanger et al., 2009), which is consistent with previous findings of health-promoting exercise habits that diminished once individuals reach adolescence (Aarts et al., 1997). This trend may be related to the gradual reduction in physical activity requirements in schools from elementary school to middle school, with greater reduction during high school and college. Third, Van Der Horst et al. (2007)'s study demonstrated that sedentary behavior habits in adolescents differed across ethnic groups, which is probably due to ethnic beliefs and habits. Fourth, Drenowatz et al. (2010); Van Der Horst et al. (2007) concluded that adolescents with lower economic and social status were more likely to exhibit lower physical activity levels and higher sedentary activity levels, which may be explained by the evidence that adolescents with higher economic levels were more likely to purchase sports equipment and had better accessibility to sports facilities (Humbert et al., 2006).

Health factors were found to affect the development of physical activity habits in adolescents. A previous study showed that normal-weight adolescents were more physically active and had more positive attitudes toward physical activity than overweight and obese adolescents (Deforche et al., 2006). This may be because overweight and obese adolescents could not maintain consistent physical activity and were unable to control their appetite and because intermittent physical activity had low effects. Further, the influence of mental health factors on physical activity habits cannot be ignored. The results of a survey indicated that adolescents who were regularly physically active had 1.49 fewer days/month (43.2%) of poor mental health compared than those who were not physically active and that all exercise types were associated with a lower mental health burden (minimum reduction of 11.8% and maximum reduction of 22.3%) (Chekroud et al., 2018).

The cognitive factors included adolescents’ perceptions of physical activity, self-concept, and body perception. Esteban-Cornejo et al.(2015) confirmed significant correlations between physical activity, cognitive performance, and self-rated health in adolescents through a systematic review (Esteban-Cornejo et al., 2015; Zhang et al., 2020). Among adolescents (14–18 years), higher perceived behavioral control, support for physical activity, and self-efficacy were associated with decreased physical activity (Craggs et al., 2011). It has been shown that physical activity and physical self-concept have a significant relationship, with perceived competence being most strongly associated with physical activity, followed by perceived fitness, general physical self-concept, and perceived physical appearance (Babic et al., 2014). Research has shown that among adolescents, boys who perceive themselves as overweight are more likely to engage in sufficient physical activity. Conversely, boys with negative body image tend to be less active compared to their peers (Kopcakova et al., 2014). Regarding the mechanisms of the influence of cognitive factors on physical activity in adolescents, it has been shown that physical activity habits can be automatically activated by situational features of frequently occurring behaviors (Aarts et al., 1997).

Lifestyle factors, including sedentary behavior, smoking, and alcohol consumption, were also found to be common factors affecting physical activity habits in adolescents. This finding is supported by extensive evidence. A systematic review revealed that physical activity habits could be established, especially when incorporated into existing lifestyles (Aarts et al., 1997). According to Vancampfort et al. (2021) engaging in leisure time sedentary behavior for at least three hours per day was associated with a 35% increase in the odds of achieving appropriate levels of moderate-to-vigorous physical activity in boys and a 22% increase in the odds in girls compared to those who engaged in sedentary activity for less than three hours per day. Milicic et al. (2019) found that regular physical activity was associated with smoking as individuals who used e-cigarettes were more likely to engage in physical activity on a regular basis than non-e-cigarette users. Pate et al. (1996) showed that low physical activity was associated with lifestyle factors, such as smoking, marijuana use, lower fruit and vegetable consumption, more television viewing, and not wearing seat belts while driving. The mechanism underlying the impact of lifestyle factors on physical activity habits among adolescents involves the automatic activation of these habits with minimal conscious effort, allowing for sustained and frequent engagement over a prolonged period (Aarts et al., 1997). Besides, it has been supported that sedentary behavior, smoking, and drinking are more likely to be adopted by adolescents than physical activity habits because physical activity requires physical effort, and laziness is human nature.

The articles included in this study were obtained by a comprehensive systematic search of major electronic databases as well as manual searches. However, it is important to acknowledge that this study is subject to several limitations. First, the literature search was restricted to English-language publications, potentially introducing language or cultural bias. Second, although we used a systematic approach to collect publicly available English-language articles, there remains a possibility of having missed relevant studies that employed different search terms. As a consequence, potential literature omissions and selection bias cannot be entirely ruled out. Lastly, the studies incorporated in this review may present substantial heterogeneity due to variations in study designs and analytical methods, which made it unfeasible to conduct a quantitative synthesis of the findings.

## 5 Conclusion

This systematic review has identified several key factors influencing the physical activity habits of adolescents. The findings indicate that boys tend to have better physical activity habits than girls, whereas girls prefer moderate-to-vigorous physical activity. Physical activity levels tend to decrease with age, and African-American adolescents have higher habitual physical activity levels than their white peers. Higher literacy levels and support from parents, teachers, friends, and others are positively associated with physical activity habits, whereas sedentary behavior, smoking, drinking, prolonged screen time, negative emotions, and new media technology are negatively associated. Additionally, higher self-efficacy and satisfaction with school sports are associated with stronger physical activity habits.

The findings of this review may serve as a reference framework and foundation for future research in this field and may also offer guidance for the development and implementation of effective interventions aimed at promoting the formation and maintenance of physical activity habits among adolescents. Future research should consider comparative studies across different countries as well as in-depth investigations into the role of habitual physical activity in improving physical and mental health indicators. Future studies should also focus on intervention and quantitative research methods to better understand the causal relationships between different factors and physical activity habits in adolescents.

## Data Availability

The original contributions presented in the study are included in the article/Supplementary Material and further inquiries can be directed to the corresponding author.
