# Supplementary material for: Correlates of physical activity habits in adolescents: A systematic review
Source: Front Physiol. 2023 Apr 21;14:1131195. doi: 10.3389/fphys.2023.1131195 (PMC10172932; doi:10.3389/fphys.2023.1131195)
Supplement: Supplementary file 1 [file Table1.DOCX]

***Table S1. Search strategy***

| Scopus=414 | (TITLE-ABS-KEY(Adolescent* OR teens OR teenager* OR juvenile OR school-aged children) AND TITLE-ABS-KEY(physical activity OR physical exercise OR sports activities OR sport movement OR sport* OR motor OR athletic sports) AND TITLE-ABS-KEY(habit* OR custom* OR Behavior habit*)) |
| --- | --- |
| Pub-med=535 | ((Adolescent*[Title/Abstract] OR teens[Title/Abstract] OR teenager*[Title/Abstract] OR juvenile[Title/Abstract] OR school-aged children[Title/Abstract]) AND (physical activity[Title/Abstract] OR physical exercise[Title/Abstract] OR sports activities[Title/Abstract] OR sport movement[Title/Abstract] OR sport*[Title/Abstract] OR motor[Title/Abstract] OR athletic sports[Title/Abstract])) AND (habit*[Title/Abstract] OR custom*[Title/Abstract] OR Behavior habit*[Title/Abstract]) |
| Web of science= 687 | ((TI=(Adolescent* OR teens OR teenager* OR juvenile OR school-aged children)) AND TI=((physical activity OR physical exercise OR sports activities OR sport movement OR sport* OR motor OR athletic sports)) AND TS=(habit* OR custom* OR Behavior habit*)) |
| EBSCOhost ERIC=18 | AB (Adolescent* OR teens OR teenager* OR juvenile OR school-aged children ) AND AB ( physical activity OR physical exercise OR sports activities OR sport movement OR sport* OR motor OR athletic sports ) AND AB ( habit* OR custom* OR Behavior habit* ) |
| Psychology and Behavioral Sciences Collection=51 | AB (Adolescent* OR teens OR teenager* OR juvenile OR school-aged children ) AND AB ( physical activity OR physical exercise OR sports activities OR sport movement OR sport* OR motor OR athletic sports ) AND AB ( habit* OR custom* OR Behavior habit* ) |

*August 14, 2022*
