# Supplementary material for: Correlates of physical activity habits in adolescents: A systematic review
Source: Front Physiol. 2023 Apr 21;14:1131195. doi: 10.3389/fphys.2023.1131195 (PMC10172932; doi:10.3389/fphys.2023.1131195)
Supplement: Supplementary file 2 [file Table2.docx]

# Table S2. Study quality evaluation and risk of bias

| **Study** | **Items** | | | | | | | | | | | | | | | | | **Score** | | **Quality** |
| --- | --- | --- | --- | --- | --- | --- | --- | --- | --- | --- | --- | --- | --- | --- | --- | --- | --- | --- | --- | --- |
|  | Q1 | | Q2 | Q3 | Q4 | Q5 | Q6 | Q7 | Q8 | Q9 | Q10 | Q11 | Q12 | Q13 | Q14 | Q15 | Q16 | Raw | % |  |
| (Bénéfice et al. 2001)  Senegal | | 1 | 1 | 1 | 1 | 0 | 1 | 1 | 1 | 1 | 1 | 1 | 1 | 1 | 1 | 1 | 0 | 14 | 87.5 | Excellent |
| (Garnier 2001)  Senegal | | 1 | 1 | 1 | 1 | 0 | 1 | 1 | 1 | 1 | 1 | 1 | 1 | 0 | 1 | 1 | 0 | 13 | 81.2 | Excellent |
| (Schmitz et al. 2002)  USA | | 1 | 1 | 1 | 1 | 1 | 1 | 1 | 1 | 1 | 1 | 1 | 1 | 1 | 1 | 1 | 1 | 16 | 100 | Excellent |
| (Gavarry et al. 2003)  France | | 1 | 1 | 1 | 1 | 1 | 1 | 0 | 1 | 1 | 1 | 1 | 1 | 0 | 1 | 1 | 0 | 13 | 81.2 | Excellent |
| (Klentrou et al. 2003)  Canada | | 1 | 1 | 1 | 1 | 1 | 1 | 1 | 1 | 1 | 1 | 1 | 1 | 0 | 1 | 1 | 1 | 15 | 93.8 | Excellent |
| (Kim et al. 2007)  Korea | | 1 | 1 | 1 | 1 | 1 | 1 | 1 | 1 | 1 | 1 | 1 | 1 | 0 | 1 | 1 | 1 | 15 | 93.8 | Excellent |
| Kaya et al. 2010  Turkey | | 1 | 1 | 1 | 1 | 1 | 1 | 0 | 1 | 1 | 1 | 1 | 1 | 0 | 1 | 1 | 1 | 14 | 87.5 | Excellent |
| (Moreno-Murcia et al. 2011)  Spain | | 1 | 1 | 1 | 1 | 1 | 1 | 1 | 1 | 1 | 1 | 1 | 1 | 0 | 1 | 1 | 1 | 15 | 93.8 | Excellent |
| (Bąk‐Sosnowska and Skrzypulec‐Plinta 2012)  Polish | | 1 | 1 | 1 | 1 | 1 | 1 | 0 | 1 | 1 | 1 | 1 | 1 | 1 | 1 | 1 | 0 | 14 | 87.5 | Excellent |
| (Hosseini et al. 2013)  Iran | | 1 | 1 | 1 | 1 | 0 | 1 | 0 | 1 | 1 | 0 | 1 | 1 | 0 | 1 | 1 | 1 | 12 | 75 | Good |
| (Piéron and Ruiz-Juan 2013)  Spain | | 1 | 1 | 1 | 1 | 1 | 1 | 0 | 1 | 1 | 1 | 1 | 1 | 0 | 1 | 1 | 0 | 13 | 81.2 | Excellent |
| (Zach et al. 2013)  Israel | | 1 | 1 | 1 | 1 | 1 | 1 | 1 | 1 | 1 | 1 | 1 | 1 | 0 | 1 | 1 | 1 | 15 | 93.8 | Excellent |
| (Dos Santos et al. 2014)  Mozambique | | 1 | 1 | 1 | 1 | 1 | 1 | 0 | 1 | 1 | 1 | 1 | 1 | 0 | 1 | 1 | 1 | 14 | 87.5 | Excellent |
| (Wafa et al. 2014)  Malaysia | | 1 | 1 | 1 | 0 | 0 | 0 | 1 | 1 | 1 | 1 | 1 | 1 | 0 | 1 | 1 | 0 | 11 | 68.8 | Good |
| (Wushe et al. 2014)  South Africa | | 1 | 1 | 1 | 1 | 1 | 1 | 0 | 1 | 1 | 1 | 1 | 1 | 0 | 1 | 1 | 1 | 14 | 87.5 | Excellent |
| (Kelishadi et al. 2016)  Iran | | 1 | 1 | 1 | 1 | 1 | 0 | 0 | 1 | 1 | 1 | 1 | 1 | 1 | 1 | 1 | 1 | 14 | 87.5 | Excellent |
| (López Sánchez et al. 2016)  Spain | | 1 | 1 | 1 | 1 | 1 | 0 | 0 | 1 | 1 | 1 | 1 | 1 | 0 | 1 | 1 | 0 | 12 | 75 | Good |
| (Carayanni et al. 2021)  Greece | | 1 | 1 | 1 | 1 | 1 | 1 | 0 | 1 | 1 | 1 | 1 | 1 | 0 | 1 | 1 | 1 | 14 | 87.5 | Excellent |
